# A Wide Dynamic Range Current Sensor Based on Torque-Mode Magnetoelectric Coupling Effect

**DOI:** 10.3390/s25237236

**Published:** 2025-11-28

**Authors:** Fuchao Li, Zihuan Huang, Yuan Meng, Yifei Zhou, Jiefu Zhang, Sujie Liu, Qiang Shi, Ziyang Ye, Lin Huang

**Affiliations:** 1 State Grid Sichuan Electric Power Company, Chengdu 610041, China; huangzh16@tsinghua.org.cn (Z.H.); yuanmeng.sgcc@outlook.com (Y.M.); zhouyf2614@126.com (Y.Z.); zhangjiefu@163.com (J.Z.); garfield7741@126.com (S.L.); dahua_224@163.com (Q.S.); vicenteyzy@hotmail.com (Z.Y.); huanglin1995@my.swjtu.edu.cn (L.H.); 2 Department of Electrical Engineering, Tsinghua University, Beijing 100084, China

**Keywords:** current sensor, magnetoelectric composite material, NdFeB/PZT, torque mode

## Abstract

The load current of the new power system has significant characteristics on a wide dynamic range, which poses challenges to current sensing technologies. This paper proposes a magnetic-sensitive element based on NdFeB/Lead Zirconate Titanate (PZT) magnetoelectric composite materials, and further develops a magnetoelectric coupling current sensor. The sensor operates in torque mode, enabling the detection of both wide dynamic range alternating currents and weak alternating currents. Experimental studies show that the sensor achieved a power-frequency current detection sensitivity of 15.56 mV/A, a linear range of (0–120) A, and a detection limit of 153 μA. The results indicate that the sensor exhibits high sensitivity in alternating current (AC) current detection, and at power frequency, possesses both a wide dynamic range and the capability to detect weak currents. Therefore, it shows great application potential in scenarios such as wide dynamic range AC current measurement and weak current detection in power systems.

## 1. Introduction

The distribution transformer area is the terminal segment of the distribution network and represents the “last mile” of electric energy delivery from generation and transmission to the user side. It carries the crucial responsibility of providing comprehensive power supply services to residential, industrial, and commercial users within the area [1,2]. Its operational status directly affects the quality and standard of the power supply service provided by grid companies. In distribution transformer areas, various types of current parameters with different characteristics exist, such as load current, zero-sequence current, capacitive current, leakage current, and residual current. The perception and measurement of various current parameters using current transformers or current sensors form the foundation for the intelligent upgrading of low-voltage transformer areas and are essential for enhancing operation and maintenance efficiency as well as improving the quality of power supply services. With the transformation of the energy structure and the evolving demands of new-type power systems, the current parameters to be measured have also given rise to sensing requirements for various unconventional current parameters. Consequently, there is a pressing need for novel current sensors capable of delivering high performance across a wide dynamic range spectrum to address these emerging challenges.

At present, various types of current sensors based on different principles have been practically applied in power systems, such as electromagnetic current transformers, shunt resistors, Hall-effect current sensors, Rogowski coils, giant magnetoresistance (GMR) current sensors, and fluxgate current sensors [3,4,5,6,7,8]. Among them, current transformer (CT) technology is the most mature and widely used. Its measurement range is limited because the core material tends to saturate under high currents [9]. Shunt resistors convert current into voltage based on Ohm’s law to enable AC current detection [10], demonstrating superior performance in low-frequency, low-current measurements. However, in high-current measurements, shunt resistors are susceptible to the effects of resistive heating. Their application scenarios are limited since they are invasive measurement methods. Hall-effect current sensors, based on the Hall effect, can detect AC currents and offer a wide measurement range in terms of amplitude and frequency [7]. However, their accuracy in detecting weak currents is relatively poor. Compared to current transformers (CTs), Rogowski coils avoid the problem of core material saturation [11], making them more suitable for wide dynamic range AC current detection. Due to their low sensitivity, they are not suitable for detecting weak currents. Fiber optic current sensors based on the Faraday magneto-optic effect offer advantages such as a wide frequency response and a large dynamic range. Nonetheless, they exhibit poor accuracy in detecting weak currents and have high costs, making them unsuitable for large-scale deployment [12]. Giant magnetoresistance (GMR) current sensors, based on the GMR effect, offer advantages such as high sensitivity, low cost, and a wide frequency range, enabling the detection of both AC and DC currents. However, when exposed to strong magnetic fields, they may suffer permanent damage and have strict requirements on operating temperature, which limits their practical applications [6,13]. Fluxgate current sensors offer advantages such as high accuracy, wide linear range, and high stability. However, due to their high production costs and stringent operating environment requirements, they are mainly used in laboratory precision instruments and are less commonly applied in field environments [8,14]. In summary, while mature technologies and products are currently available for detecting steady-state high currents at power frequency, ongoing research is necessary to address the detection of weak currents and non-steady-state currents.

Against this background, it remains necessary to continuously develop current sensors based on new materials and novel principles to meet the demands of specialized applications. In recent years, novel sensor devices based on the magnetoelectric coupling effect have attracted widespread attention. The magnetoelectric coupling effect refers to the mutual coupling between magnetization and polarization, achieved by fabricating magnetostrictive materials and piezoelectric materials into magnetoelectric composites. When a sensing element based on magnetoelectric composite materials is placed in an alternating magnetic field, it can generate a voltage signal output. Therefore, this principle can be utilized to detect magnetic fields and currents. In 2005, Dong et al. developed a current sensor based on a circular trilayer PZT/Terfenol-D/PZT magnetoelectric composite, capable of detecting currents in the range of 1 Hz to 100 kHz, with a measurement range of (2–10.8) A [15]. In 2013, Zhang et al. developed a ring-shaped current sensor with a Terfenol-D/PZT/NdFeB structure, capable of detecting currents in the range of 10 Hz to 40 kHz, with a sensitivity of 10 mV/A [16]. However, this kind of magnetoelectric coupling sensor, which uses Terfenol-D as the magnetic material layer, invariably requires the application of a DC bias magnetic field of several hundred to several thousand Oersteds. Overall, most existing magnetoelectric sensors face significant limitations in distribution transformer area applications. Their typical requirement for an additional DC bias magnetic field increases system complexity and infrastructure overhead, while their inherently narrow dynamic range and low sensitivity are inadequate for monitoring the widely fluctuating currents encountered in distribution networks. These shortcomings collectively undermine measurement accuracy and operational practicality in this specific scenario, highlighting the critical need for the alternative approach developed in this work.

In response to the limitations of conventional magnetoelectric sensors, this paper proposes a novel current sensor based on an NdFeB/PZT composite. Unlike previous Terfenol-D-based designs that require a substantial DC bias, our sensor operates on a magneto–mechanical–electrical coupling principle in torque mode, thereby eliminating the need for an external bias magnet. This novel design simultaneously provides high sensitivity across a wide dynamic range, including exceptional capability for weak current detection. These combined attributes demonstrate its distinct potential for accurate load and leakage current detection in distribution transformer areas.

## 2. Design and Fabrication of the Sensing Element

**Figure 1:**
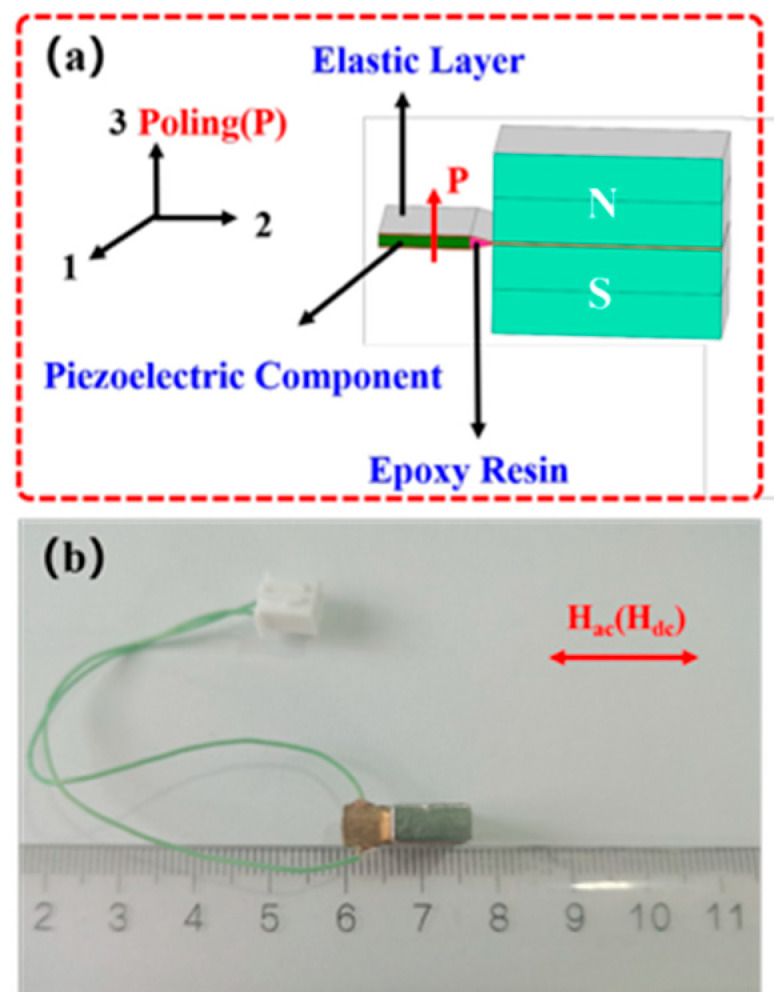
NdFeB/PZT sensing element: (**a**) structural schematic; (**b**) photograph of the element.

In this study, the magnetoelectric sensing element was fabricated using NdFeB/PZT magnetoelectric composite materials, where the NdFeB serves as the permanent magnet material with a remanent magnetization of 1.01 T, and the PZT is the piezoelectric material of type PZT-5H. The main parameters of the PZT material are the piezoelectric coefficients d_33_ = 593 pC/N and d_31_ = −274 pC/N, and the elastic compliance S_11_^E^ = 16.5 × 10^−12^ m^2^/N. The sensitive element is designed with a cantilever beam structure, as shown in Figure 1a. In this structure, the piezoelectric component is sandwiched by an elastic layer, while the other end of the elastic layer is clamped by a pair of NdFeB permanent magnets. Upon exposure to an external AC magnetic field, the permanent magnet material experiences a torque, which in turn induces compressive stress in the piezoelectric material. The piezoelectric material then generates an electrical signal output, thereby enabling passive sensing of the external AC magnetic field. The piezoelectric component, elastic layer, and permanent magnets are bonded together using epoxy resin, which is cured at room temperature for 24 h. The fabricated sensing element is shown in Figure 1b. Its total length is less than 15 mm, which facilitates installation within the air gap of a magnetic concentrator ring.

According to the torque principle, the sensing element experiences a torque under an applied magnetic field, and the magnitude of the torque can be calculated by Equation (1) [17]:

(1) $$M_{0}=\mu \times B\approx \left(B_{r}\cdot V\right)\times B,$$

where *M*_0_ represents the torque, μ is the magnetic moment, *B* is the external magnetic flux density, *B_r_* is the remanent flux density of the permanent magnet, and *V* is the total volume of the magnet. As can be seen from Equation (1), all parameters except *B* are constants; therefore, the magnitude of *M*_0_ is determined solely by *B*, and the two exhibit a linear relationship.

Based on the analysis of the magneto–mechanical–electrical coupling relationship of the sensing element, the correlation between the sensor output voltage *V*_3_ and the torque *M*_0_ can be further derived, as expressed in Equation (2) [18]:

(2) $$V_{3}=\frac{M_{0}\omega^{2}l^{2}md_{33}}{6\bar{E}IC_{0}},$$

where *V*_3_ is the ME voltage of piezoelectric layer. *ω* is the angular velocity, which is calculated as *ω* = 2*πf*. *f* is the frequency of AC magnetic field. *l* is the length of the cantilever structure. *m* is the mass of cantilever structure, including the tip mass. *d*_33_ is the piezoelectric coefficient. $\bar{E}$ is the equivalent elastic modulus of the cantilever. *I* is the inertia moment, and *C*_0_ is the capacitance of the piezoelectric layer. Therefore, it can be inferred that the output voltage *V*_3_ exhibits a linear correlation with the magnetic flux density *B*. Accordingly, the magnetic flux density *B* of an external magnetic field can be determined by measuring the output voltage *V*_3_ of the sensing element.

**Figure 2:**
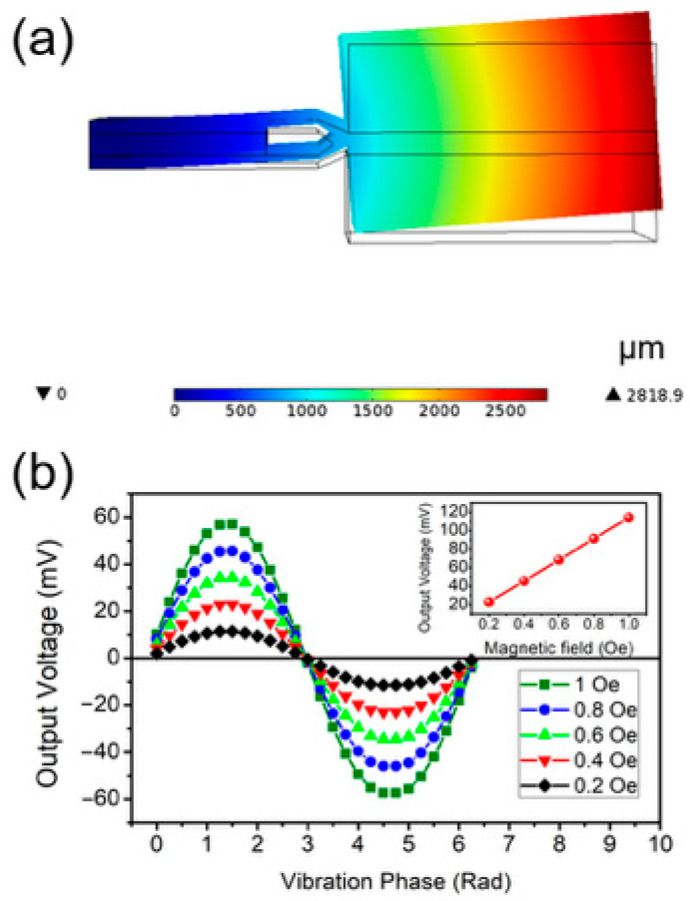
Finite element simulation results: (**a**) deformation of the sensing element under an external magnetic field; (**b**) variation of the output voltage of the sensing element with respect to the external magnetic field strength.

In this study, the designed sensing element was simulated using the COMSOL finite element simulation software (v. 5.5.) to investigate the variation pattern of its output signal under changes in the external magnetic field. The simulation results are presented in Figure 2. As shown in Figure 2a, when subjected to an external magnetic field excitation, the sensing element undergoes a bending deformation, which induces stress in the piezoelectric material and consequently generates an output signal via the piezoelectric effect. When the frequency of the applied magnetic field matches the resonant frequency of the sensing element, the maximum tip displacement of the element can reach 2.8189 mm. Figure 2b illustrates the output signals of the sensing element under sinusoidal alternating magnetic fields with root mean square (RMS) values of 0.2 Oe, 0.4 Oe, 0.6 Oe, 0.8 Oe, and 1 Oe. The simulation results indicate that the sensing element can effectively track the waveform of the alternating magnetic field, and the amplitude of the output voltage V3 exhibits a linear correlation with the amplitude of the applied magnetic field B. The simulation outcomes are in good agreement with the theoretical derivations given in Equations (1) and (2), demonstrating the high feasibility of employing the sensing element in this magnetic torque mode for magnetic field detection.

**Figure 3:**
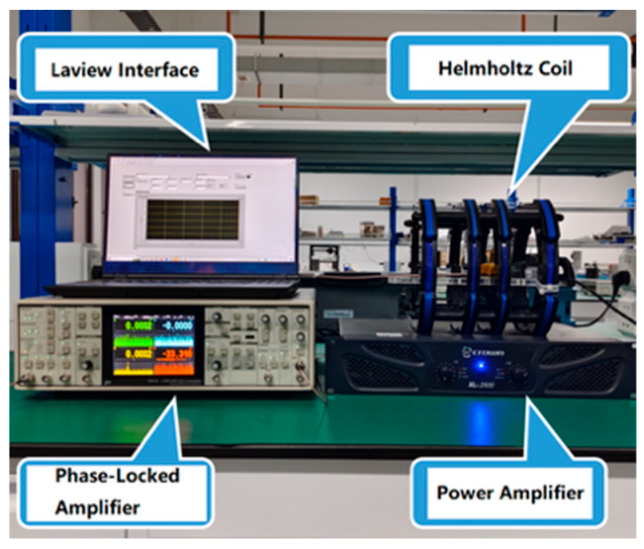
Test environment for evaluating the detection capability of sensitive elements to alternating and static magnetic fields.

**Figure 4:**
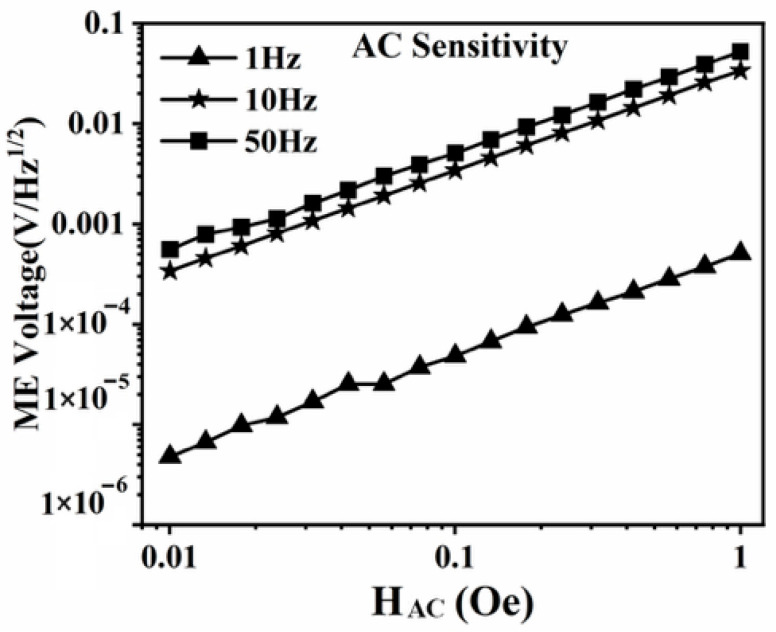
Output of sensitive elements under alternating magnetic fields.

The experimental setup, as illustrated in Figure 3, was established to validate the capability of the designed sensing element in detecting alternating magnetic fields. In the experiments, a magnetic field generation device composed of a power amplifier and a Helmholtz coil was employed to produce alternating magnetic fields with frequencies of 1 Hz, 10 Hz, and 50 Hz, and amplitudes ranging from 0.01 Oe to 1 Oe. The fabricated sensing element was placed within the generated magnetic field, and its output voltage was measured using an SR865A lock-in amplifier (Stanford Corporation, Stamford, CT, USA). During the measurements, the lock-in amplifier was configured with a time constant of 1 s and a slope of 24 dB/oct, corresponding to an equivalent noise bandwidth (ENBW) of 78.125 mHz. The device output voltage was normalized by the square root of the ENBW. The experimental results, shown in Figure 4, indicate that the output voltage of the device exhibited a linear correlation with the applied alternating magnetic field, in agreement with both the theoretical analysis and simulation results. Furthermore, the results demonstrate that the detection limit of the sensing element for alternating magnetic fields is 0.01 Oe.

The above experimental results are consistent with the theoretical analysis and simulation results, indicating that the sensitive element designed in this work can be used for detecting alternating magnetic fields and can be further developed into a current sensor for AC current measurement.

## 3. Design and Development of a Magnetoelectric Coupling Current Sensor

**Figure 5:**
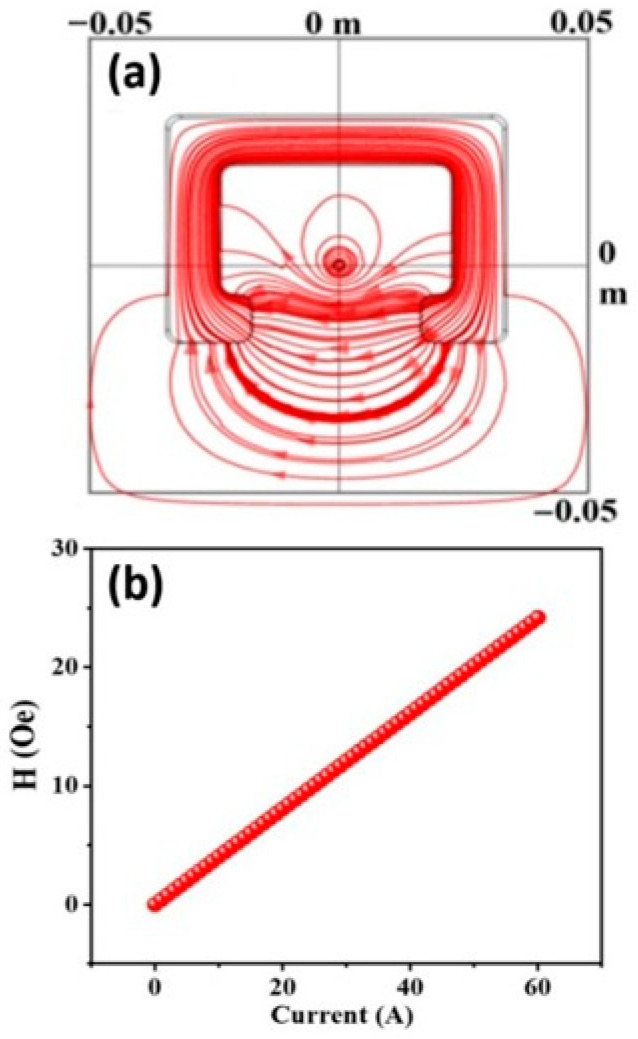
Simulation of the magnetic concentrator ring structure: (**a**) magnetic field distribution under the test current; (**b**) relationship between the magnetic flux density in the air gap and the amplitude of the test current.

In current sensor design, a magnetic concentrator ring with an air gap is commonly used to concentrate the magnetic field, with the sensitive element placed in the air gap for magnetic field detection, thereby enabling overall current measurement. In this study, the structure of the magnetic concentrator ring was modeled and analyzed using COMSOL finite element simulation software to investigate its structural optimization, as illustrated in Figure 5. Figure 5a shows that the magnetic field generated by the current under test in the conductor is concentrated by the magnetic concentrator ring and transformed into a relatively uniform magnetic field in the air gap. The center point of the air gap in the magnetic concentrator ring was taken as the reference point for magnetic field calculation, from which the relationship between the magnetic flux density and the current amplitude was obtained. As shown in Figure 5b, the magnetic flux density in the air gap exhibited a linear relationship with the current amplitude. By continuously optimizing the parameters of the simulation model, the optimal design of the magnetic concentrator ring was determined, resulting in a rectangular structure with dimensions of 64 mm × 47 mm × 20 mm, an air gap width of 32 mm, and made of 1J85 permalloy.

**Figure 6:**
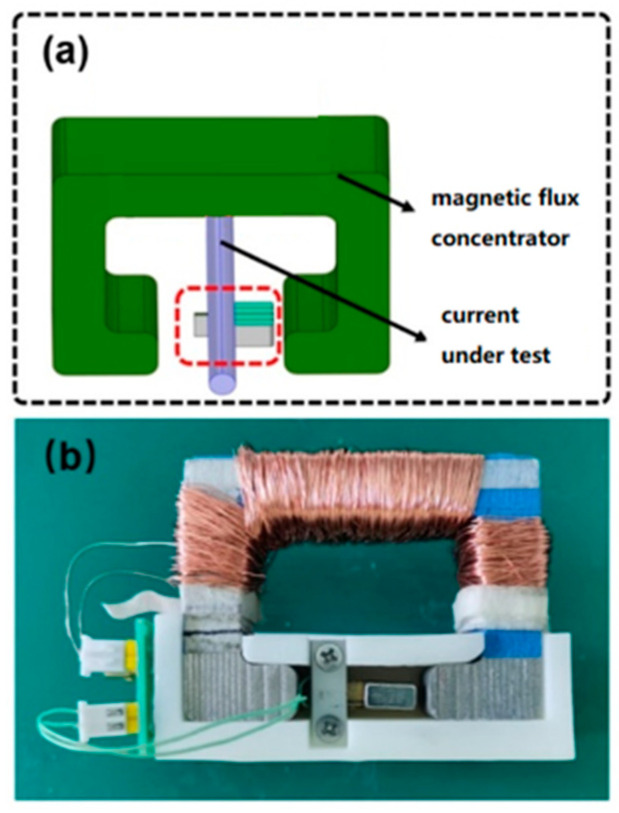
Magnetoelectric current sensor: (**a**) structural diagram; (**b**) physical sensor.

In this study, a magnetoelectric coupling current sensor was further designed by placing the previously described magnetoelectric coupling sensitive element in the air gap of a magnetic concentrator ring, as shown in Figure 6a. The fabricated prototype of the sensor, based on this design, is shown in Figure 6b, where the magnetic concentrator ring and the sensitive element are mounted on a plastic base. The sensor has two sets of signal ports: one for the sensor output voltage and the other for the excitation signal.

## 4. Performance Study of Magnetoelectric Coupled Current Sensors

**Figure 7:**
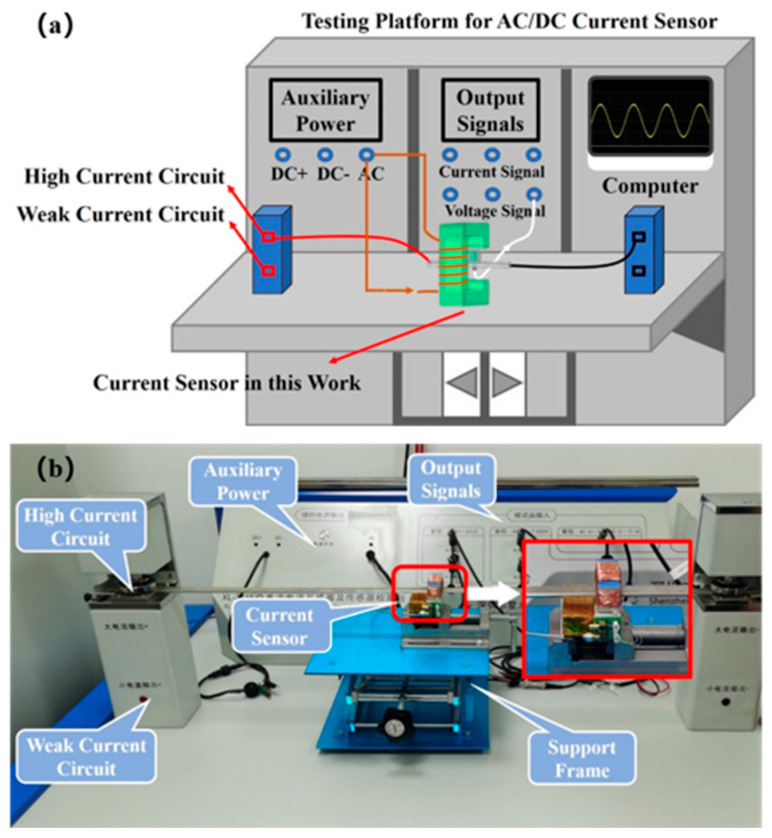
(**a**) Schematic diagram of the testing platform for the AC/DC current sensor. (**b**) Photo of the experimental setup.

In this work, the performance of the developed magnetoelectric coupled current sensor was experimentally investigated using a current sensor test platform. The schematic diagram and the actual experimental setup are shown in Figure 7a and Figure 7b, respectively. The test platform was equipped with both a high-current test circuit and a low-current test circuit, capable of providing alternating currents up to 600 A and 1 A, respectively. It was also furnished with a current calibrator and a measuring instrument with an accuracy class of 0.05.

**Figure 8:**
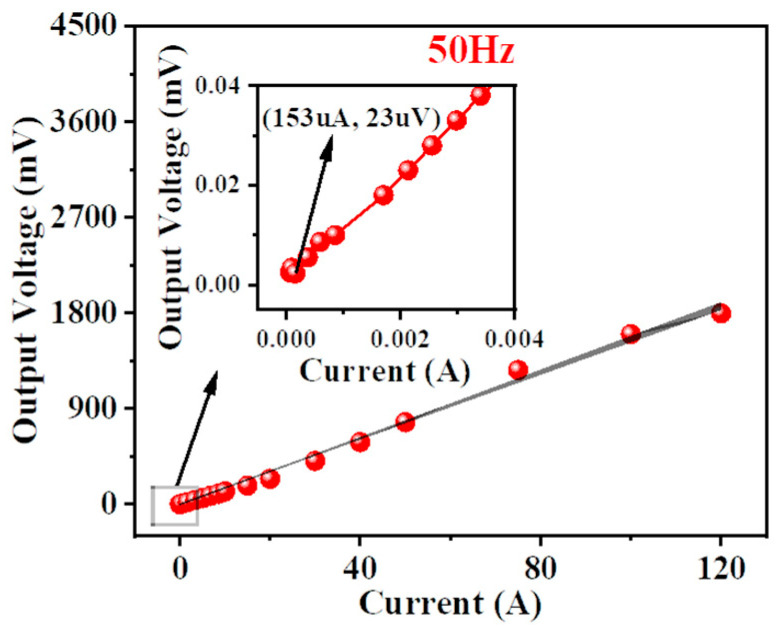
The testing results for 50 Hz AC current in the range of 0–120 A.

The output voltage was measured at 39 points across the 0–120 A range, and a fitting curve was plotted. The test results of the magnetoelectric coupled current sensor for the power-frequency currents are shown in Figure 8. When the RMS value of the measured power-frequency current varied from 0 A to 120 A, the sensor output voltage exhibited a linear response with respect to the current. Based on the slope of the fitted curve, the power-frequency current sensitivity of the sensor was calculated to be approximately 15.56 mV/A. The inset in Figure 8 shows the measurement results under small currents, from which the detection limit of the sensor for power-frequency currents was estimated to be approximately 153 μA.

## 5. Conclusions

In this work, a magnetoelectric (ME) sensitive element was designed and fabricated based on the magneto–mechanical–electric coupling effect of the NdFeB/PZT composite material in torsion mode. A magnetoelectric coupled current sensor was then developed, and its performance was evaluated using a current sensor test platform. The experimental results show that the developed ME coupled current sensor exhibited a power-frequency current sensitivity of 15.56 mV/A, a linear range of 0–120 A, and a detection limit of 153 μA. The results indicate that the sensor possesses both high sensitivity and a wide dynamic range, enabling it to detect large currents at the hundred-ampere level while maintaining a detection limit down to the hundred-microampere level.

In conclusion, this study lays the groundwork for a new class of magnetoelectric current sensors based on the torque-mode coupling mechanism. To advance this technology toward practical implementation, subsequent work will systematically investigate the sensor’s frequency response, operational bandwidth, and resilience to external magnetic interference, exploring its applicability in diverse scenarios.

## Data Availability

The original contributions presented in this study are included in the article. Further inquiries can be directed to the corresponding author.

## Abbreviations

The following abbreviations are used in this manuscript:

GMR	Giant Magneto Resistance
CT	Current Transformer
AC	Alternating Current
RMS	Root Mean Square
ME	Magnetoelectric
ENBW	Equivalent Noise Bandwidth
